# FetNet: a recurrent convolutional network for occlusion identification in fetoscopic videos

**DOI:** 10.1007/s11548-020-02169-0

**Published:** 2020-04-29

**Authors:** Sophia Bano, Francisco Vasconcelos, Emmanuel Vander Poorten, Tom Vercauteren, Sebastien Ourselin, Jan Deprest, Danail Stoyanov

**Affiliations:** 1 Wellcome/EPSRC Centre for Interventional and Surgical Sciences (WEISS) and Department of Computer Science, University College London, London, UK; 2 Department of Mechanical Engineering, KU Leuven University, Leuven, Belgium; 3 School of Biomedical Engineering and Imaging Sciences, King’s College London, London, UK; 4 Department of Development and Regeneration, University Hospital Leuven, Leuven, Belgium

**Keywords:** Deep learning, Surgical vision, Twin-to-twin transfusion syndrome (TTTS), Fetoscopy, Video segmentation, Computer assisted interventions (CAI)

## Abstract

**Purpose:**

Fetoscopic laser photocoagulation is a minimally invasive surgery for the treatment of twin-to-twin transfusion syndrome (TTTS). By using a lens/fibre-optic scope, inserted into the amniotic cavity, the abnormal placental vascular anastomoses are identified and ablated to regulate blood flow to both fetuses. Limited field-of-view, occlusions due to fetus presence and low visibility make it difficult to identify all vascular anastomoses. Automatic computer-assisted techniques may provide better understanding of the anatomical structure during surgery for risk-free laser photocoagulation and may facilitate in improving mosaics from fetoscopic videos.

**Methods:**

We propose FetNet, a combined convolutional neural network (CNN) and long short-term memory (LSTM) recurrent neural network architecture for the spatio-temporal identification of fetoscopic events. We adapt an existing CNN architecture for spatial feature extraction and integrated it with the LSTM network for end-to-end spatio-temporal inference. We introduce differential learning rates during the model training to effectively utilising the pre-trained CNN weights. This may support computer-assisted interventions (CAI) during fetoscopic laser photocoagulation.

**Results:**

We perform quantitative evaluation of our method using 7 in vivo fetoscopic videos captured from different human TTTS cases. The total duration of these videos was 5551 s (138,780 frames). To test the robustness of the proposed approach, we perform 7-fold cross-validation where each video is treated as a hold-out or test set and training is performed using the remaining videos.

**Conclusion:**

FetNet achieved superior performance compared to the existing CNN-based methods and provided improved inference because of the spatio-temporal information modelling. Online testing of FetNet, using a Tesla V100-DGXS-32GB GPU, achieved a frame rate of 114 fps. These results show that our method could potentially provide a real-time solution for CAI and automating occlusion and photocoagulation identification during fetoscopic procedures.

**Electronic supplementary material:**

The online version of this article (10.1007/s11548-020-02169-0) contains supplementary material, which is available to authorized users.

## Introduction

Twin-to-twin transfusion syndrome (TTTS) is a fetal anomaly affecting $10{-}15\%$ of identical twins sharing a monochorionic placenta [3]. It is caused by the presence of abnormal placental vessels that disproportionately transfuse blood from one twin to the other. The recipient of excessive blood is at risk of heart failure, while the twin with insufficient circulation will have a deficient growth. If left untreated, mortality is above 90% [3] for both twins. Laser therapy for TTTS can be done before birth through fetoscopy to significantly increase survival (between 68 and 85% [4]). Fetoscopic treatment of TTTS aims at photocoagulating the abnormal vessels in the placenta using a fetoscopic camera with a retractable laser ablation tool in its working channel, interrupting the undesired blood transfusion from one twin to the other [20]. The technique has many challenges related to poor visibility [15], varying placenta position [8], and vessel identification [18] that can cause some target vessels to be missed [16]. To safely perform photocoagulation, the surgeon requires a clear view of the placenta and a clear path between the ablation tool and the target vessels. This can be challenging in numerous situations as the fetoscopic visibility can significantly degrade with scene depth changes due to defocus, amniotic fluid turbidity, and high variability in scene illumination. Additionally, the larger recipient twin is freely moving, causing unpredictable and frequent occlusions of the laser ablation tool. Automatic detection and display of fetoscopic events may help the surgeon in navigating through the placenta, identifying abnormal vessels, and alerting for potential risks such as insufficient or unsafe laser ablation. Fetoscopic event detection can also allow a more effective retrospective case analysis by retrieving meaningful event timestamps from long raw videos.

Deep learning architectures have shown promising results in temporal analysis of laparoscopic videos for tool detection and tracking, and surgical phase identification [5, 14, 24]. The primary focus of surgical workflow analysis is to identify sequential, non-overlapping phases where each phase usually lasts for several minutes. On the contrary, fetoscopic events are unordered, overlapping (multiple labels can co-exist, e.g. “tool” and “occlusion” ), and can occur in multiple instances, and within very short periods of times. These factors make it unfeasible to directly incorporate the temporal constraints from [5, 14, 24], such as phase relationship priors encoding in the hidden-Markov models (HMM) and temporal smoothing, into fetoscopic event identification.

In the case of TTTS laser therapy, deep learning methods have only been marginally explored. Ablation detection through a ResNet encoder has been proposed to create timelines of the surgical procedure [25]. The method [25] also inferred when the surgeon is ready for ablating the target vessel, and however, this is only evaluated qualitatively. Overall, research efforts towards computer-assisted TTTS therapy have mostly focused on creating navigation maps of the placental vessels through mosaicking techniques [2, 6, 17, 23]. While promising results have been obtained for short-term sequences, the mosaicking techniques do not scale well for longer duration videos. Temporal fetoscopic event segmentation could provide additional context for navigation and mapping algorithms using the fetoscopic camera, which is the only source of information currently available in the operating room.

In this paper, we address the automatic detection of events that determine photocoagulation conditions. Specifically, we propose a recurrent convolutional network termed as FetNet for spatio-temporally identifying fetoscopic events such as clear view, occlusions, ablation tool visibility, and vessel ablation in fetoscopic videos. The proposed FetNet performs event prediction in an online manner by integrating a convolutional neural network (CNN) and a long short-term memory recurrent neural network (LSTM-RNN) for simultaneously encoding both spatial (single frame) and temporal (multi-frame) information. This type of architecture has not been utilised previously for fetoscopic video analysis, where fetoscopic videos pose additional challenges for network training due to the dominant (sometimes abrupt) camera movements, floating amniotic fluid particles, and dynamically changing views. FetNet extends and improves previous work on ablation detection [25] by additionally identifying clear view, occlusion and ablation tool presence events and improving performance through the use of a recurrent network for multi-frame analysis. We show through 7-fold cross-validation and comparison that FetNet performs significantly better due to joint encoding of both spatial and temporal dependencies in an end-to-end network. We obtain an online prediction rate of 114 fps which shows the potential of using FetNet during live fetoscopy.

**Fig. 1:**
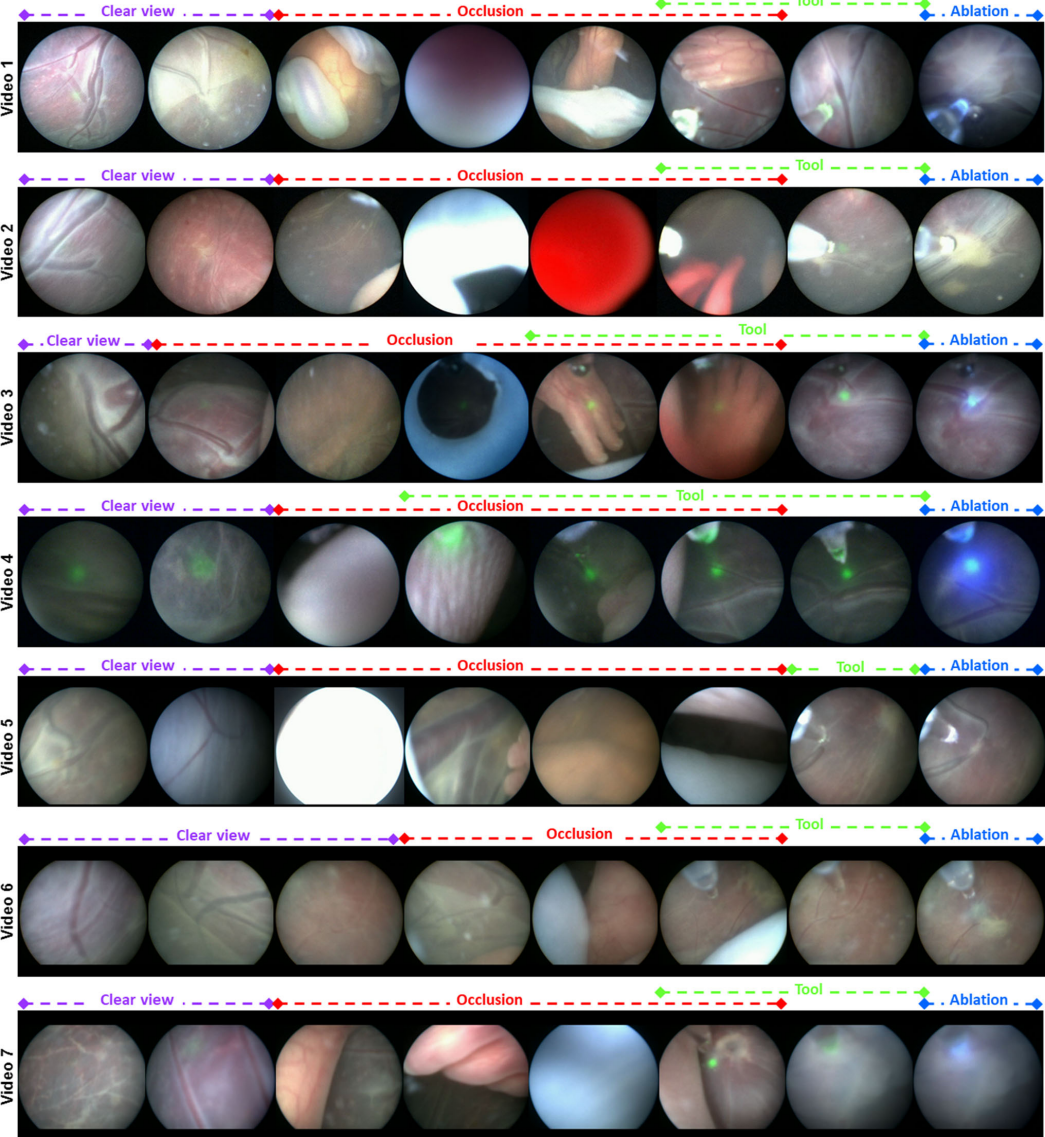
Representative cropped images from the seven fetoscopic videos used in our experiments displaying the four multi-label event classes

## Problem formulation

Given a fetoscopic sequence $\mathbf {F}= \{F_t\}_{t=1}^N$, where *N* is the sequence’s length, we are interested in predicting the fetoscopic event labels for each frame $F_t$. We introduce four event labels, namely clear view, occlusion, ablation tool presence and vessel ablation (representative images shown in Fig. 1). *Clear view*: The placenta surface is visible in the fetoscopic field-of-view (FoV) without the presence of any occlusion. *Occlusion*: The fetoscopic FoV is partially or completely blocked due to the presence of fetus, heavy amniotic fluid particles or fetoscopic port. The FoV may also get occluded when the fetoscope comes very close to the placenta or fetus, often causing defocus or strong light reflection. *Tool*: The ablation tool is present in the FoV of the fetoscope. This event is identified by the presence of an opaque notch (tool head) and projection of green light circle on the placenta surface. *Ablation*: The vessel photocoagulation is being performed. This event is identified by the presence of the ablation tool, projection of blue light due to increased intensity of the laser and changes in the appearance of the targeted vessels resulting in disconnected vessels. While clear view and rest of the three labels are mutually exclusive, tool and (sometimes) ablation may occur along with occlusion. Hence, fetoscopic event identification is considered to be a multi-label classification problem.

**Fig. 2:**
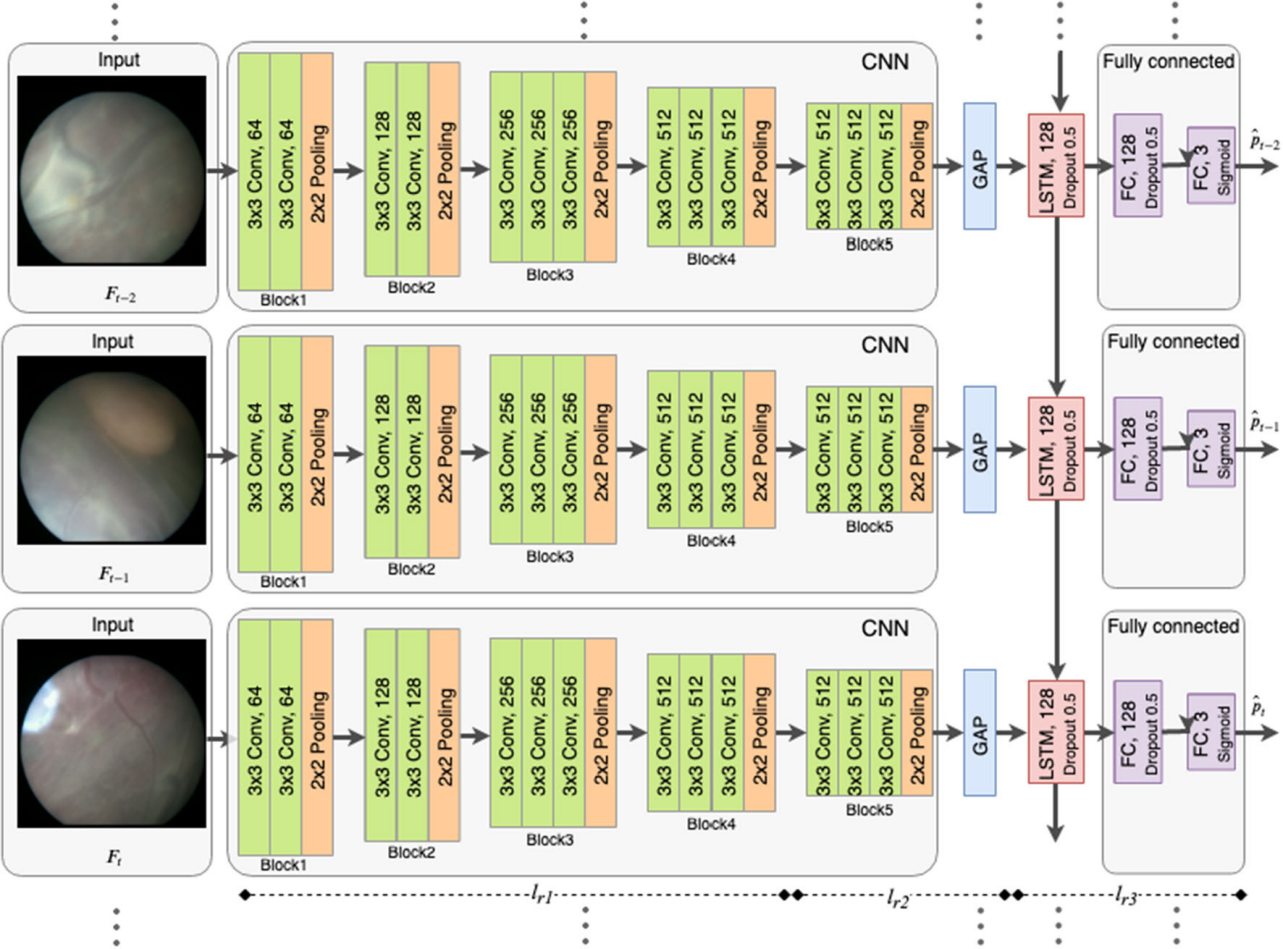
An overview of the proposed FetNet for event classification in fetoscopic videos. Spatial representation of each frame is encoded by a CNN (VGG16 architecture) while the temporal representation is encoded using LSTM followed by fully connected layers. Differential learning rate is applied during network training

## Proposed methodology

We present a deep learning framework to solve the multi-label spatio-temporal classification problem in fetoscopic videos. The proposed FetNet architecture is shown in Fig. 2. We use a CNN to extract discriminative visual features from each frame and utilised an LSTM-RNN to model the temporal information in consecutive frames. The CNN and LSTM-RNN networks are integrated to form an end-to-end recurrent convolutional network such that the complementary information of the visual and temporal features can be sufficiently encoded for accurate classification.

### Spatial information encoding

For spatial information encoding, we use the VGG16 [21] network as the backbone. Due to its simplistic architecture which introduces more convolutional layers with smaller filters resulting in increased depth of the network and network performance. The network consists of an input ($224\times 224\times 3$), 13 convolutional layers and 5 max-pooling layers (Fig. 2, with filters having a receptive field of $3\times 3$, a stride of 1 and same spatial padding for preserving the spatial resolution after convolution. Max pooling is performed after some of the convolutional layers over a $2\times 2$ pixel window with a stride of 2 to down-sample the input representation. Block5 (Fig. 2) of VGG16 outputs $7\times 7\times 512$ dimensional feature maps which are then fed into a global average pooling (GAP) layer. GAP computes the average output of each feature map in the last layer to significantly reduce the data. This results in a 512-dimensional visual descriptor that forms the input of the LSTM layer for temporal information encoding.

### Temporal information encoding

Sequential data in fetoscopic videos carry contextual information for event category identification. For example, an ablation event is enclosed within the tool event. Likewise, during fetoscopic environment exploration in the initial phase of the procedure, clear view interlaces with the occlusion category. Jointly learning of such spatio-temporal relationships can provide better prediction compared to learning from only spatial (single frame) data.

Our proposed network utilises LSTM [10] to encode temporal information since LSTMs are robust for learning temporal dependencies. We use sequence-to-sequence configuration of LSTM, which has been used extensively for sequential data modelling (e.g. sample-to-sample activity recognition [9], machine translation [1, 22]). In our proposed network, the visual descriptor from GAP forms the input of the LSTM layer having hidden layers with 128 units. The fetoscopic procedure can last for several minutes (e.g. 30 min of video has 45,000 frames). Retaining the memory for the complete duration of the procedure is not resource effective. Therefore, time frames (TF) are defined within which the information of past frames are retained and are reset in every new TF. The LSTM layer is followed by a fully-connected (FC) layer with 128 units and an output layer with 3 units with sigmoid activation. It is possible to have multiple possible labels at each frame as the labels are not mutually exclusive, and therefore, sigmoid activation is used to get independent output probabilities. For training, we use binary cross-entropy loss,

1 $$\begin{aligned} L(p,\hat{p})= \frac{1}{N}\sum _{t=0}^{N}\left( p_t\log (\hat{p}_t)+(1-p_t)\log (1-\hat{p}_t) \right) \end{aligned}$$

penalising each output unit independently. $\hat{p}_t$ is the predicted output and $p_t$ is the ground-truth and *N* is the total number of samples. We train the FetNet on *Occlusion*, *Tool* and *Ablation* event categories. A clear view, $\hat{p}^\mathrm{cv}_t$, occurs when none of the other events ($\hat{p}^\mathrm{occlusion}_t,\hat{p}^\mathrm{tool}_t,\hat{p}^\mathrm{ablation}_t$) exist, and its output at frame *t* is computed as

2 $$\begin{aligned} \hat{p}^{cv}_t = 1 - max(\hat{p}^\mathrm{occlusion}_t,\hat{p}^\mathrm{tool}_t,\hat{p}^\mathrm{ablation}_t). \end{aligned}$$

### Differential learning

Using pre-trained model weights for transfer learning gives early convergence with better performance compared to when training a model from scratch [12]. Differential learning implies having a smaller learning rate for earlier layers and relatively larger learning rate for the latter ones. This is useful because the latter layers are initialised randomly and need significant updates compared to earlier layers that are initialised with the pre-trained weights [26]. We use this concept for training our network. The spatial encoder layers are initialised using the pre-trained ImageNet [7] weights and the remaining layers are initialised using Xavier initialisation [11]. We split our network into three parts (as indicated in Fig. 2), i–e, split 1 is from Block1 to Block4, split2 is from Block4 to GAP and split 3 is from LSTM to FC (output). We then applied a smaller learning rate, $l_{r1}= 10^{-7}$, on the first split and gradually increased learning rates of $l_{r2}=10^{-5}$ and $l_{r3}=10^{-3}$ on the second and third splits, respectively.

### Training details

We present implementation details of the VGG16 (fine-tuned) and FetNet architectures. For fine-tuning, the VGG16 architecture on our dataset, the FC layers are modified with output units of 2048, 512 and 3, and the model is initialised with the pre-trained ImageNet weights. A dropout of 0.2 is applied to the FC layers, and a learning rate of $5e^{-5}$ and Adam optimiser with a momentum of 0.9 is used. During FetNet end-to-end training (Fig. 2), we use the ImageNet pre-trained weights for the CNN module, randomly initialised the remaining weights using [11], and use differential learning rates (Sect. 3.3) with Adam optimiser. A dropout of 0.2 is used for the latter layers to avoid over-fitting. A TF of 150 frames (equivalent to 12 s of video) is used for the LSTM. Note that this TF is selected such that it is larger than the maximum duration of an ablation event; ablation events are of the shortest interval compared to other events. This ensured that the TF is neither too small that it misses covering the temporal dynamics of the sequence nor too large to limit the GPU resources.

Data augmentation is performed at run-time as this allows adding relatively more variations compared to a fixed size preprocessed augmentation set. Augmentation is performed by applying rotation, horizontal and vertical flip, and illumination intensity change. We randomly (with a 50% chance) augment each frame in every epoch during VGG16 fine-tuning. Likewise, augmentation is applied to each TF in every epoch during FetNet training. During training, we use early stopping, i.e., if the validation loss does not decrease any further in the latest 5 epochs (iterations), the training is terminated. Due to early stopping, the training generally lasted between 50 and 120 epochs. The network weights that gave the best validation accuracy before early stopping are used for testing. The use of validation dataset and early stopping ensured that we are not over-fitting the model. We perform 7-fold cross-validation on left-out (unseen) test videos to demonstrate the robustness of our model. Our framework is implemented in Keras with TensorFlow backend using a single Tesla V100-DGXS-32GB GPU of an NVIDIA DGX-station.

## Experimental analysis

### Dataset description

**Table 1:** Distribution of fetoscopic videos (in frames) for each event label

Video#	Resolution	#Frames	Clear view	Occlusion	Tool	Ablation
Video 1	$470\times 470$	25,900	5604	13,167	7903	3163
Video 2	$540\times 540$	17,030	5643	2439	8877	1498
Video 3	$550\times 550$	12,000	1896	6570	6846	306
Video 4	$570\times 570$	17,450	1886	5227	12,113	1273
Video 5	$570\times 570$	22,000	9370	2900	7336	2458
Video 6	$550\times 550$	27,000	8020	7474	10,155	5064
Video 7	$550\times 550$	17,400	8328	8112	2522	156
Total		138,780	40,747	45,889	55,752	13,891

We perform validation of the proposed FetNet using seven in vivo fetoscopic sequences (138,780 frames) acquired at 25 fps with a display resolution of $720\times 576$ *pixels*. Each video captured a separate real operation performed on a different patient. We cropped each video into a square to remove the black left and right margins of the display. As a result, the cropped frame resolution is different in each sequence due to different settings of the fetoscope in each case (Table 1). All frames are resized to $224\times 224$ to form the input to the FetNet. Some representative frames from our dataset are shown in Fig. 1. When observing frames from a particular class across different videos in Fig. 1, we can notice the intra-class variability due to different visual appearance, changing lighting, and changing views due to moving fetoscope. For example, the clear view within a sequence can have a significantly different appearance. Though there is no tool present in video 4 (clear view) yet a strong tool light is visible making such frames very similar to the tool class. Occlusion blocks a significant amount of the FoV most of the times but sometimes only cover a very small region (as visible from video 2 and video 6 frames) making it very similar to the clear view category. Such light occlusions are usually observed when transiting from the clear view to the occlusion category. The operating port can also occlude the view (video 3). Tool and ablation are interlacing events and can sometimes have a very similar appearance (video 5 and video 6).

We manually annotated the in vivo fetoscopic sequences for the four event categories using the video labeller application.^1^ A single annotator first annotated these sequences, which were then verified by a clinician. The definition of each category as detailed in Sect. 2 is used for creating the annotation labels. When manually annotating sequential data, it is easier for a human annotator to distinguish between tool and ablation due to the ablation light intensity and colour change. Moreover, these two categories are interlacing with each other. Likewise, occlusion and clear view are interlacing for most of the duration of the video. A clear view transition into occlusion as soon as the placental view is even slightly blocked by the presence of fetus, umbilical cord, operating port or tool. Creating multi-label frame-level annotations for 138,780 frames is a time-consuming task and took on average 10 h to annotate a single fetoscopic sequence. The distribution of each label, in terms of the number of frames, is reported in Table 1.

### Evaluation protocol

For the experimentation, all fetoscopic sequences are subsampled at 12.5 fps giving us a total of 69,390 frames from the seven fetoscopic videos. We perform 7-fold cross-validation, wherein each fold training is performed using 6 videos and testing is performed using the left-out (unseen) video. The training set is sub-divided into the train (5 videos) and validation (the shortest video from the training set) sets. The validation set is needed during training to avoid over-fitting (as discussed in Sect. 3.4). An input frame size of $224\times 224$ is used for all methods under analysis.

To quantitatively evaluate the performance of our proposed method, we report the precision, recall and F1-score at a chosen operating point (obtained by thresholding the output probabilities at 0.5 for each event category). We also report the precision-recall curves, area under the curve (AUC) and bar plots for each event category for all 7-folds.

### Comparison methods

We compare FetNet, with and without differential learning, against an existing ablation detection [25] and three CNN-based [21] methods similar to the one proposed in  [5]. VGGFE_SVM used features extracted from the VGG16 for training a support vector machine [5], VGG16_fine is the fine-tuned network (detailed in Sect. 3.4) and VGG_temporal applied temporal smoothing to VGG16_fine predictions. Details about the configuration of different methods under comparison are reported in Table 2. All methods mentioned are initialised with the pre-trained ImageNet weights, since through experimentation we found that random initialisation always resulted in comparatively lower performance. An ablation detection method using histogram feature extraction and support vector machine was also tested in [25]. However, the reported results are significantly worse than the deep learning-based methods, and therefore we do not include it in our experimental comparison.

**Table 2:** Configuration details of different methods under comparison

Method	Network details	Learning rate
Ablation_detect [25]	ResNet50 [13] with binary output layer for ablation detection	Fixed to $10^{-5}$
VGGFE_SVM [5]	Features from the VGG16 [21] FC2 layer classified using SVM	–
VGG16_fine [21]	VGG16 network with FC layers having 2048, 512 and 3 units	Fixed to $10^{-6}$
VGG16_temporal [5]	Temporal averaging with a median filter of size 6 (samples) applied to the VGG16 predictions	–
FetNet_noDL	Proposed FetNet (Fig. 2) without differential learning	Fixed to $10^{-6}$
FetNet_DL	Proposed FetNet (Fig. 2) with differential learning	$l_{r1}=10^{-7}$, $l_{r2}= 10^{-5}$, $l_{r3}=10^{-3}$

All networks are initialised with the pre-trained ImageNet weights

**Table 3:** 7-fold cross-validation results of the proposed FetNet and its comparison with the existing methods

Method	Class					
		Clear	Occlusion	Tool	Ablation	Average
Ablation_detect [25]	Precision	–	–	–	0.81	0.81
	Recall	–	–	–	0.71	0.71
	F1-score	–	–	–	0.76	0.76
VGGFE_SVM	Precision	0.52	0.55	0.68	0.32	0.52
	Recall	0.42	0.70	0.50	0.19	0.45
	F1-score	0.46	0.62	0.58	0.24	0.47
VGG16_fine	Precision	0.66	0.69	0.76	0.96	0.77
	Recall	0.47	0.69	0.73	0.61	0.63
	F1-score	0.55	0.69	0.74	0.75	0.68
VGG16_temporal	Precision	0.72	0.70	0.76	0.96	0.79
	Recall	0.46	0.68	0.73	0.56	0.61
	F1-score	0.56	0.69	0.74	0.71	0.68
FetNet_noDL	Precision	0.72	0.70	0.86	0.95	0.81
	Recall	0.78	0.60	0.90	0.69	0.74
	F1-score	0.74	0.65	0.88	0.80	0.77
FetNet_DL	Precision	0.86	0.69	0.92	0.96	0.86
	Recall	0.84	0.79	0.94	0.95	0.88
	F1-score	0.85	0.74	0.93	0.95	0.87

### Results and discussion

**Fig. 3:**
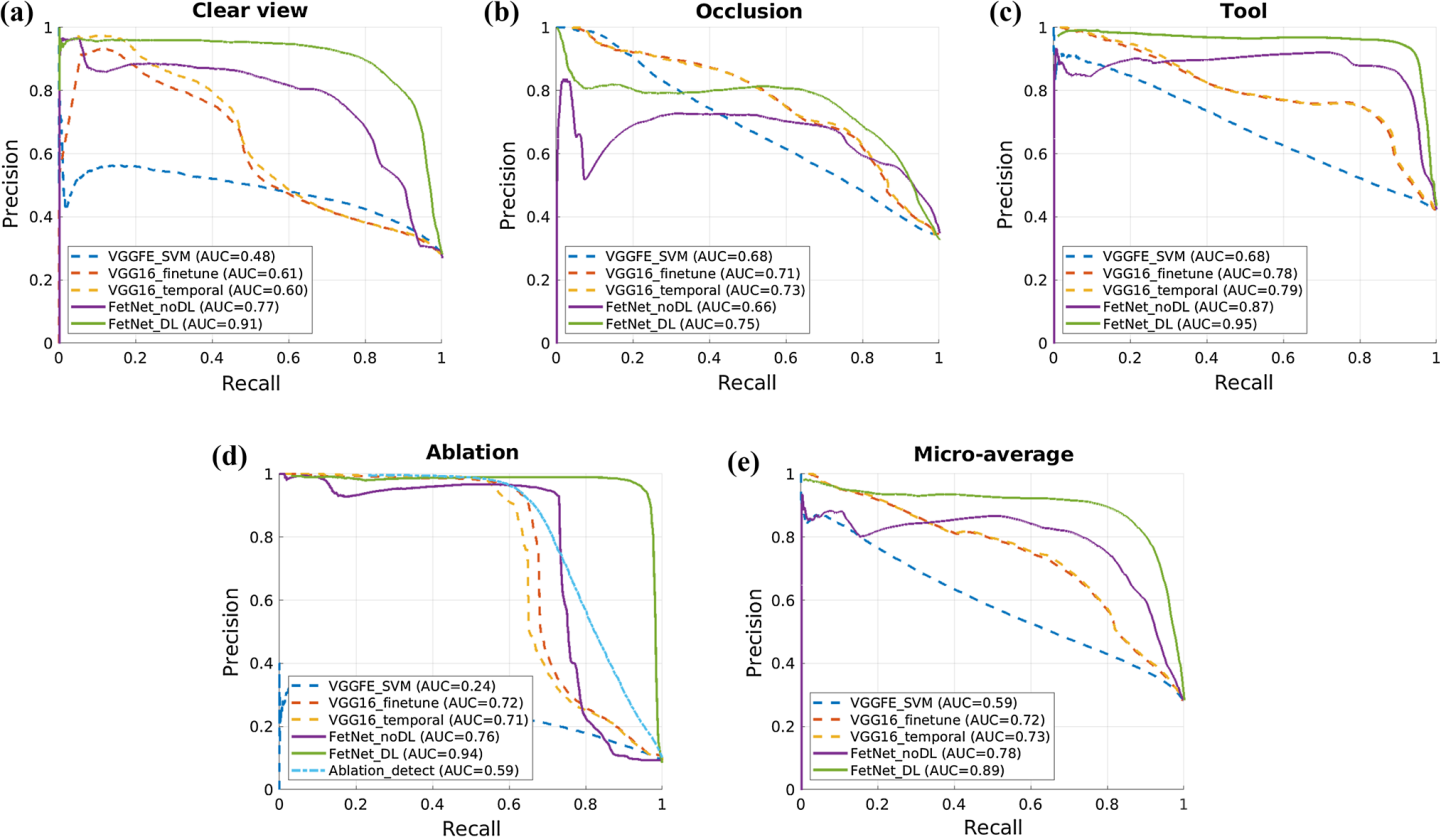
Precision-recall curves along with AUCs of different methods under comparison for **a** clear view, **b** occlusion, **c** tool and **d** ablation classes. **e** The micro-average precision-recall over all the classes

**Fig. 4:**
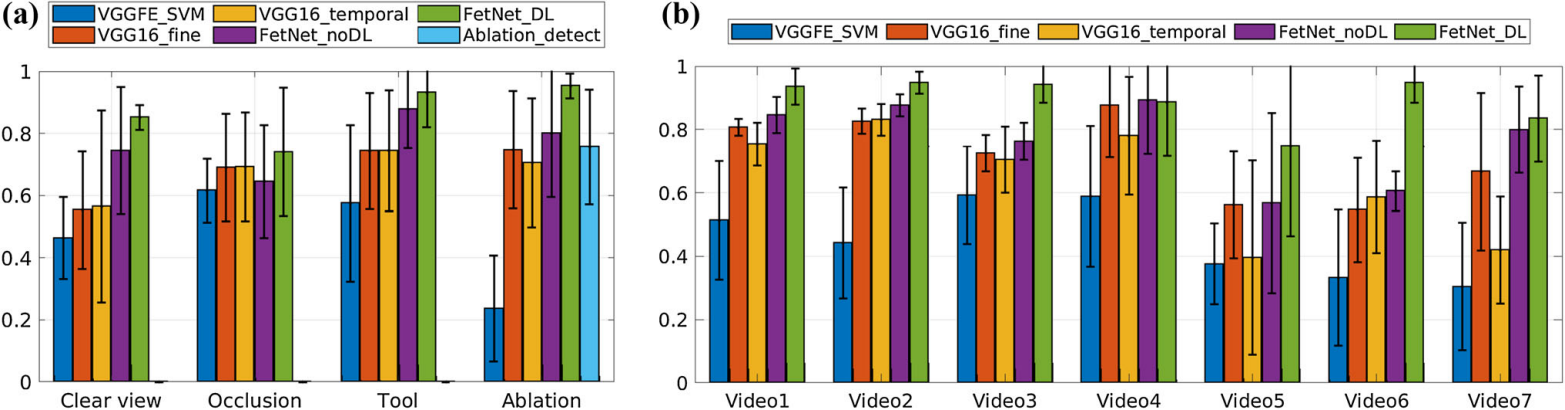
Performance comparison of different methods. F1-scores and standard deviations; **a** over 7-folds for each event; **b** over 4 events for each fold

Table 3 reports the results of the 7-fold cross-validation, while the corresponding precision-recall curves along with the AUCs are presented in Fig. 3. Bar plots of F1-scores for each event category and fold are reported in Fig. 4. We start by comparing the results of spatial-only methods. Ablation_detect gave an overall F1-score of 0.76 for the ablation event, which is comparable to the ablation event of VGG16_fine with an F1-score of 0.75. VGGFE_SVM performed poorly compared to all other spatial-only methods which show that using CNN features alone is not sufficient. End-to-end parameter learning and fine-tuning is needed for adapting the pre-trained CNN features to the distribution of new data.

Next, we analyse the effect of adding temporal constraints. Introducing temporal filtering VGG16_temporal [5] in VGG16_fine helped in improving the performance of clear view and occlusion events but resulted in decreased performance for the tool and ablation events; because the instances of clear view and ablation events are of much longer duration compared to the tool and ablation events. In surgical phase recognition [5, 14, 24], applying temporal smoothing helps since the phases are generally of longer duration and sequential. However, this is not the case in fetoscopic event detection. We observe from Table 3 and Fig. 3 that the proposed FetNet outperformed other methods due to joint spatial and temporal encoding. The use of differential learning helped in further improving the performance along with early convergence of the model compared to FetNet_noDL. FetNet_DL resulted in an overall performance gain of 19% over VGG16_fine and VGG16_temporal, and of 10% over FetNet_noDL.

The performance of each event category can be observed from the precision-recall curves in Fig. 3 and the bar plots of F1-scores (along with the standard deviation over the 7-folds) in Fig. 4a. Note that occlusion has a lower F1-score of 0.74 (AUC = 0.75) compared to the clear view (0.85, AUC = 0.91), tool (0.93, AUC = 0.95) and ablation (0.95, AUC = 0.94) for the proposed FetNet_DL because of the intra-class variability and transition frames. There are frames in which occlusion only spans over $2{-}3\%$ of the FoV, hence making it challenging to classify such frames at a resolution of $224\times 224$ pixels. Compared to the transition frames, partial or heavy occlusion frames are more accurately classified. Likewise, tool and ablation have a significantly different visual appearance with less variability compared to the occlusion. Hence, the tool and ablation classes are inferred with high confidence as depicted in Figs. 3c, d and 4a. The overall superior performance of FetNet_DL (AUC = 0.89) is further verified from Fig. 3e which shows the micro-averaged precision-recall curves of the methods under comparison.

**Fig. 5:**
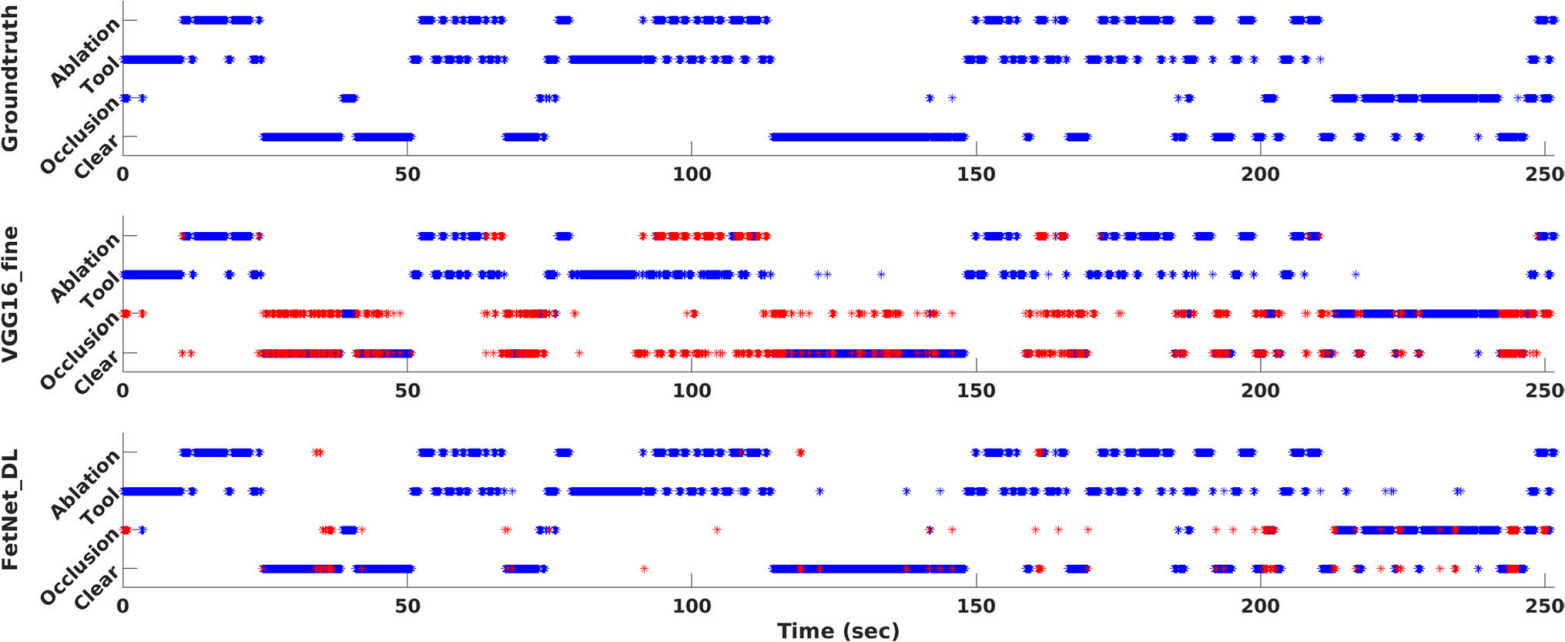
A snapshot of timeline showing predictions for video 1. Groundtruth (top) and correct predictions from VGG_fine (middle) and FetNet_DL (bottom) are shown in blue. The erroneous predictions are shown in red

Figure 4b shows the bar plots of the F1-scores for each video (fold) and the standard deviation over the 4 classes. Video 5 and 6 are the most challenging ones with a significantly different appearance (as evident from Fig. 1) compared to other videos and contain fetoscopic lens artefacts (creating a honeycomb-like pattern over each frame). Likewise, video 7 frames are out of focus resulting in blur artefacts, hence contributing to its relatively lower performance. Nevertheless, we can observe from Fig. 4b that the proposed FetNet with differential learning helped in significantly improving the prediction results. Video 4 is an interesting case, where the F1-scores of VGG_fine (0.88), FetNet_noDL (0.89) and FetNet_DL (0.89) are comparable, the reason being the low illumination, highly visible tool and ablation events (Fig. 1), and 76% of frames belonging to the tool and ablation events (Table 1); thus resulted in predictions with high accuracy. Figure 5 shows the comparison of FetNet_DL (bottom) and VGG16_fine (middle) on a snapshot of timeline. VGG16_fine struggled in correctly predicting events on consecutive frames and heavily misclassified clear view as occlusion while fluctuating a lot from one class to the other. On the other hand, FetNet_DL resulted in much better predictions with fewer erroneous classifications. A supplementary video is included with this paper depicting the visualisation of the qualitative results.

The online prediction rate of FetNet_DL is recorded to be 114 fps, which is higher than Ablation_detect (106 fps) and VGG16_fine (70 fps). This is because FetNet_DL has fewer network parameters (15,059,779) compared to Ablation_detect (23,589,761) and VGG16_fine (67,147,587). Since we use LSTM with sequence-to-sequence configuration (mentioned in Sect. 3.2), there is no delay in the prediction. This shows the potential of using FetNet in real-time clinical systems. Overall, the proposed FetNet outperformed existing methods due to the additional temporal information encoding, which shows that such a network may serve as a useful tool for assisting and automating occlusion and photocoagulation identification during fetoscopic procedures. Moreover, FetNet can provide additional context for navigation and mapping algorithms using the fetoscopic camera and can help in generating better mosaics from fetoscopic videos [2] by focusing only on frames with occlusion-free views. FetNet can also assist in designing in vivo fetoscopic vessel segmentation [19] strategies by initially focusing only on occlusion-free frames. Additionally, vessel segmented prediction masks can be utilised for generating vascular mosaics (an expanded FoV image of the placental vascular structure) which may support the identification of abnormal vessels during the TTTS laser therapy.

## Conclusion

We proposed a recurrent convolutional network for fetoscopic event identification in in vivo fetoscopic videos. Our proposed FetNet architecture jointly encoded the spatial (single frame) and temporal (multi-frame) cues by integrating the VGG16 architecture with LSTM-RNN in an end-to-end manner. We evaluate by performing 7-fold leave-one-out cross-validation on 7 in vivo fetoscopic videos. We showed that FetNet outperformed the existing methods and better encodes the spatial and temporal dependencies when trained using a pre-trained on ImageNet with differential learning rates on the four-event classes. Qualitative results showed that the proposed FetNet better handled the challenges in the fetoscopic environment and resulted in improved prediction for multi-label frames compared to the other methods. This showed that unlike spatial-only encoding methods, a spatio-temporal encoding method is better suited for video data analysis. Additionally, FetNet is able to operate at a high frame rate, which suggests that such a network could be integrated into real-time systems.

## Electronic supplementary material

Below is the link to the electronic supplementary material.

Supplementary material 1 (mp4 8598 KB)

## Footnotes

1 Video labeller from Matlab https://uk.mathworks.com/help/vision/ref/videolabeler-app.html: Last accessed—07/04/2020.
